# Repetitive transcranial magnetic stimulation elicits weight loss and improved insulin sensitivity in type 2 diabetic rats

**DOI:** 10.1002/ame2.12483

**Published:** 2024-10-22

**Authors:** Xuanjin Chen, Ruru Wang, Xin Wang, Ming Liu, Zhipeng Liu, Tao Yin, Chen Li

**Affiliations:** ^1^ Tianjin Key Laboratory of Biomedical Materials Institute of Biomedical Engineering Chinese Academy of Medical Sciences & Peking Union Medical College Tianjin China

**Keywords:** fatty acid synthesis and metabolism, insulin sensitivity, rTMS, type 2 diabetes, weight loss

## Abstract

**Background:**

Type 2 diabetes (T2D) accounts for the majority of diabetes incidences and remains a widespread global chronic disorder. Apart from early lifestyle changes, intervention options for T2D are mainly pharmaceutical.

**Methods:**

Repetitive transcranial magnetic stimulation (rTMS) has been approved by the FDA as a therapeutic intervention option for major depressive disorders, with further studies also indicating its role in energy metabolism and appetite. Considering its safe and non‐invasive properties, we evaluated the effects of rTMS on systemic metabolism using T2D rats.

**Results:**

We observed that rTMS improved glucose tolerance and insulin sensitivity in T2D rats after a 10‐day exposure. Improved systemic insulin sensitivity was maintained after a 21‐day treatment period, accompanied by modest yet significant weight loss. Circulating serum lipid levels, including those of cholesteryl ester, tryglyceride and ceramides, were also reduced following rTMS application. RNA‐seq analyses further revealed a changed expression profile of hepatic genes that are related to sterol production and fatty acid metabolism. Altered expression of hypothalamic genes that are related to appetite regulation, neural activity and ether lipid metabolism were also implicated.

**Conclusion:**

In summary, our data report a positive impact of rTMS on systemic insulin sensitivity and weight management of T2D rats. The underlying mechanisms via which rTMS regulates systemic metabolic parameters partially involve lipid utilization in the periphery as well as central regulation of energy intake and lipid metabolism.

## Graphical abstract

Repetitive transcranial magnetic stimulation (rTMS) can significant improve insulin sensitivity in rats with type 2 diabetes, accompanied by weight loss. The beneficial effects of rTMS are partly attributable to a central‐to‐peripheral regulatory action of rTMS on hepatic lipid metabolism, a novel finding which is worth further exploration.

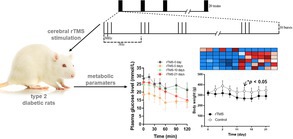

## 1 INTRODUCTION

Type 2 diabetes (T2D) is a chronic metabolic disorder, attributable to the worsening of pancreatic β‐cell dysfunction as well as insulin resistance.^1^, ^2^, ^3^ T2D accounts for over 90% of all‐case diabetes and the current therapeutic approaches for clinical T2D intervention mainly include lifestyle changes, prescription of anti‐diabetics and insulin replacement therapy.^1^, ^2^, ^3^ Despite the rapid development of anti‐diabetics and insulin analogues, the alarmingly increasing rate of the diabetic population vastly aggravates demand for anti‐diabetics and insulin production, especially in less developed countries.^1^, ^2^, ^3^

Transcranial magnetic stimulation (TMS), implemented based on Faraday's law of electromagnetic induction, can generate induced electrical currents through time‐varying magnetic fields.^4^ Three main types of TMS patterns have been reported, including single‐pulse TMS, paired‐pulse TMS, and repetitive‐TMS (rTMS). While single‐pulse and paired‐pulse TMS stimulation tends to be instantaneous, rTMS, which relies on patterned pulses with specific frequencies, has been shown to either inhibit or excite sustained cortical neuron activities.^5^, ^6^, ^7^ Since rTMS can stimulate brain neuron activity and has been proven to be relatively safe and non‐invasive, controlled rTMS delivery has been investigated as a potential intervention therapy for various neurological and psychiatric diseases. Indeed, the US Food and Drug Association (FDA) has approved the use of rTMS systems for the treatment of major depressive disorders.^8^ Moreover, later research has also shown that high‐frequency rTMS acting on the dorsolateral prefrontal cortex could reduce appetite and body weight in obese individuals.^9^, ^10^

Glucose sensing neurons located in regions such as the hypothalamus, brainstem, and substantia nigra within the central nervous system can directly perceive changes in peripheral glucose levels, integrate glucose homeostasis signals, and regulate peripheral glucose metabolism.^11^ Exploring the potential of the non‐invasive rTMS intervention on glucose or metabolic regulation may provide a safe and cost‐effective option for T2D prevention. Furthermore, considering that rTMS at high frequency has been reported to cause changes in regions related to metabolic regulation such as the hypothalamus and pituitary,^12^ and its implied effects on metabolic disorders, we have investigated the potential effects of high‐frequency rTMS on metabolism using T2D rats.

## 2 METHODS

### 2.1 Experimental reagents and animals

Streptozotocin (STZ), sodium citrate, glucose, insulin and PBS were obtained from Sigma‐Aldrich, Beijing. The Magstim Rapid2 stimulator and 8‐shaped coil were obtained from Mastic, Whitland, UK. The experimental animals used here were from the Laboratory Animal Centre of the Academy of Military Medical Sciences (Beijing, China). The protocols involved in the animal experiments performed in this study were designed and approved by the Animal Ethics and Welfare Committee of the Chinese Academy of Medical Sciences (Approval No. IRM‐DWLL‐2020001) and performed in strict accordance with the Guidelines for Use and Care of Laboratory Animals of the Chinese Academy of Medical Sciences.

### 2.2 Induction of T2D

Male Wistar rats (4 weeks old, ~150 g) were maintained on a high‐fat diet (HFD; 60% fat content) for 8 weeks. Hyperglycemia was then induced by a total of 3–5 intraperitoneal (i.p.) injections of 80 mg/kg streptoxolocin (STZ) per injection given every other day. Plasma glucose levels were measured from the tail blood using a OneTouch Ultra glucometer (Lifescan, Johnson & Johnson, CA). The animals were considered diabetic when the random plasma glucose levels were over 11.1 mmol/L for 3 consecutive days.

### 2.3 Experimental design

A total of 12 hyperglycemic rats were randomly divided into 2 groups, the rTMS‐treated group (rTMS group), and a pseudo‐stimulation sham (Control) group. A total of 3 sessions of rTMS were performed. Each session involved daily application of high‐frequency rTMS for 5 consecutive days followed by 2 non‐stimulation days. Both the rTMS‐treated and the sham Control groups were subjected to an i.p. glucose tolerance test (IPGTT) and an i.p. insulin tolerance test (IPGTT) every 7 days. Tissue samples of the liver and hypothalamus were extracted at the end of experiments and used for RNA‐seq analysis. Serum samples were also collected for ELISA and lipidomic analysis. In addition, liver samples were processed and sectioned for H&E and Oil red O staining.^13^ Rat pancreata were also extracted, fixed and sectioned for immunofluorescence staining by following previously reported procedures.^14^

### 2.4 rTMS stimulation procedure

**FIGURE 1:**
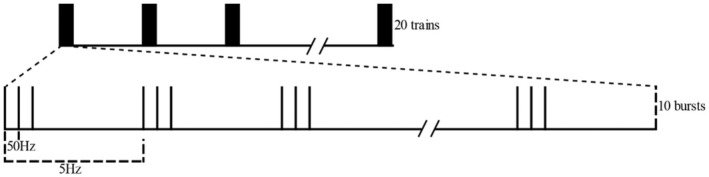
Schematic representation of the iTBS stimulation trains.

The Magstim Rapid2 stimulator and Magstim 8‐coil were used for cerebral neuron stimulation. The figure‐of‐eight‐shaped coil consists of two circular coils with an inner diameter of 8 mm and an outer diameter of 30 mm, and the intersection point of the two circular coils is the center point of the figure‐of‐eight‐shaped coil. During the stimulation process, the coil was fixed on a scaffold in order to alleviate the pressure on the rats as a result of the coil weight. The coil was placed parallel to the parietal bone and approximately 2 mm away from the scalp. The center point of the coil was aligned with the center point of the sagittal suture. The rats were held and kept conscious during the rTMS procedure. A total of 20 intermittent theta burst stimulation (iTBS) sequences were performed with a repetition period of 10 s per rat per day. Each stimulation sequence contained 10 pulse clusters with a repetition rate of 5 Hz, and each pulse cluster contained 3 stimulation pulses with a repetition rate of 50 Hz. The magnetic induction intensity of the stimulation pulse was 30% of the maximum output intensity of the stimulator (approximately 350 mT), as shown in Figure 1. A total of 5 sessions were conducted per rat per week. For the sham control group, the coil was placed approximately 8 cm away from the rat brain, the sound of the stimulation was simulated, although no actual electromagnetic stimulation was triggered.

Intraperitoneal glucose tolerance test (IPGTT) and intraperitoneal insulin tolerance test (IPITT).

Rats were fasted for 8 h prior to the IPGTTs or IPITTs. For the IPGTT, a glucose solution with a concentration of 0.2 g/mL (dissolved in PBS) was i.p. injected at a dose of 2 g/kg BW. For the IPITT, a insulin solution with a concentration of 0.1 IU/mL was injected at 0.5 IU/kg BW (dissolved in citrate sodium buffer). Plasma glucose levels were measured from the rat tail veins at 0, 15, 30, 45, 60, 90, and 120 min following glucose or insulin injection. Food intake and body weight of each rat were also recorded every week.

### 2.5 Statistical analysis

All data were expressed as mean ± standard error of mean (s.e.m.), and Graphpad Prism 9.0 was used for statistical analysis. Differences with a *p* value below 0.05 were considered statistically significant. For multiple group comparison, multiple factor analysis of variance (ANOVA) was used, and post hoc multiple validation was performed. For group comparison between 2 groups, a standard Student's *t*‐test was used. ImageJ (1.52i Wayne Rasband) was used for image analysis.

## 3 RESULTS

rTMS stimulation improved systemic insulin sensitivity and reduced average body weight of T2D rats.

**FIGURE 2:**
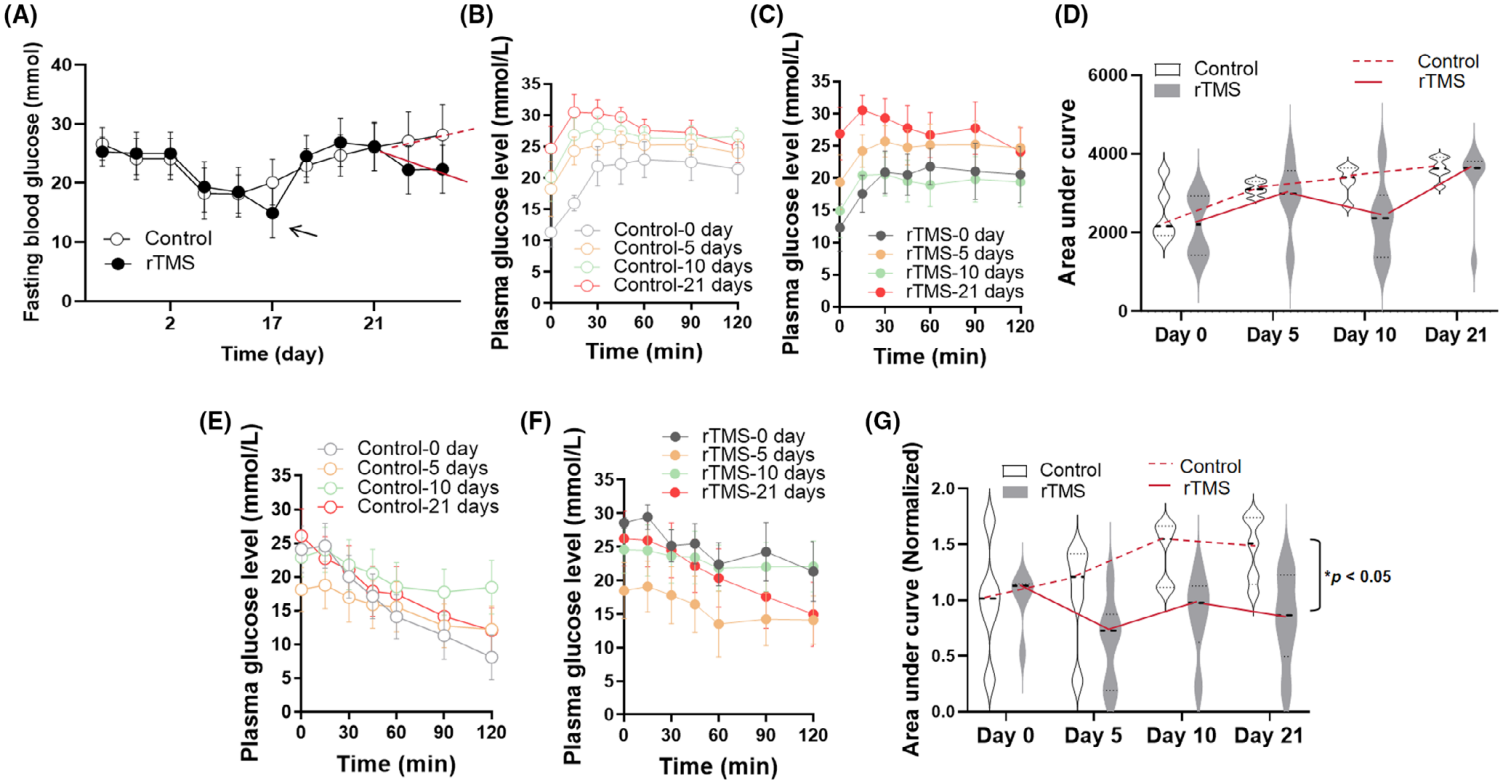
(A) Random blood glucose levels records of the rTMS‐treated (black solid circles) and the Control (open circles) groups of T2D rats. (B and E) Results of IPGTT (B) and IPITT (E) from the Control group at Day 0 (open gray circle), Day 5 (open yellow circle), Day 10 (open mint circle) and Day 21 (open red circle). (C and F) Results of IPGTT (C) and IPITT (F) from the rTMS group at Day 0 (solid gray circle), Day 5 (solid yellow circle), Day 10 (solid mint circle) and Day 21 (solid red circle). (D and G) Area under curve (AUC) of the IPGTT (D) and IPITT (G) data from both rTMS (gray shaded areas and solid red line) and Control (gray outlined areas and dashed red line) groups. All data are presented as mean ± s.e.m., *n* = 6.

As shown in Figure 2A, rTMS stimulation exerted no significant impact on fasting plasma glucose of T2D rats. However, a moderate decrease between Day 10 and Day 15 during rTMS intervention was observed (Figure 2A, black arrow). In addition, toward the end of the 21‐day stimulation procedure, fasting plasma glucose in the sham Control group tended to increase (Figure 2A, red dashed line), whereas for the rTMS group, the fasting plasma glucose levels exhibited a decreasing trend, as highlighted in Figure 2A (red solid line).

Regarding systemic glucose tolerance, exacerbated glucose intolerance was observed in the sham Control group with time, shown as different colored open circles relative to the gray open circles indicating Day 0 (Figure 2B). For groups that received rTMS, although glucose intolerance was not completely reversed, amelioration of glucose intolerance was recorded at Day 10 (Figure 2C, mint solid circle), and remained comparable to Day 0 (Figure 2C, shown as lowered mint solid circles compared to the gray solid circles). This is also observable in the area under the curve (AUC) analysis of Figure 2D, where a clear difference in glucose tolerance was shown at Day 10 (Figure 2D, highlighted by red solid line).

In contrast, rTMS did significantly improve systemic insulin sensitivity in T2D rats (Figure 2E,F). As expected, for the sham Control group, systemic insulin sensitivity worsened progressively from Day 0 (Figure 2E, gray open circle) to Day 21 (Figure 2E, red open circle), shown by the colored circles mostly sitting above the gray open circles. For the rTMS‐treated group, however, insulin sensitivity improved most noticeably on Day 5 (Figure 2F, yellow solid circle). Moderate improvement was also observed on Day 10 (Figure 2E, red solid circle) and the AUC analysis confirmed the significant effects of rTMS treatment on systemic insulin sensitivity in T2D rats (Figure 2G).

**FIGURE 3:**
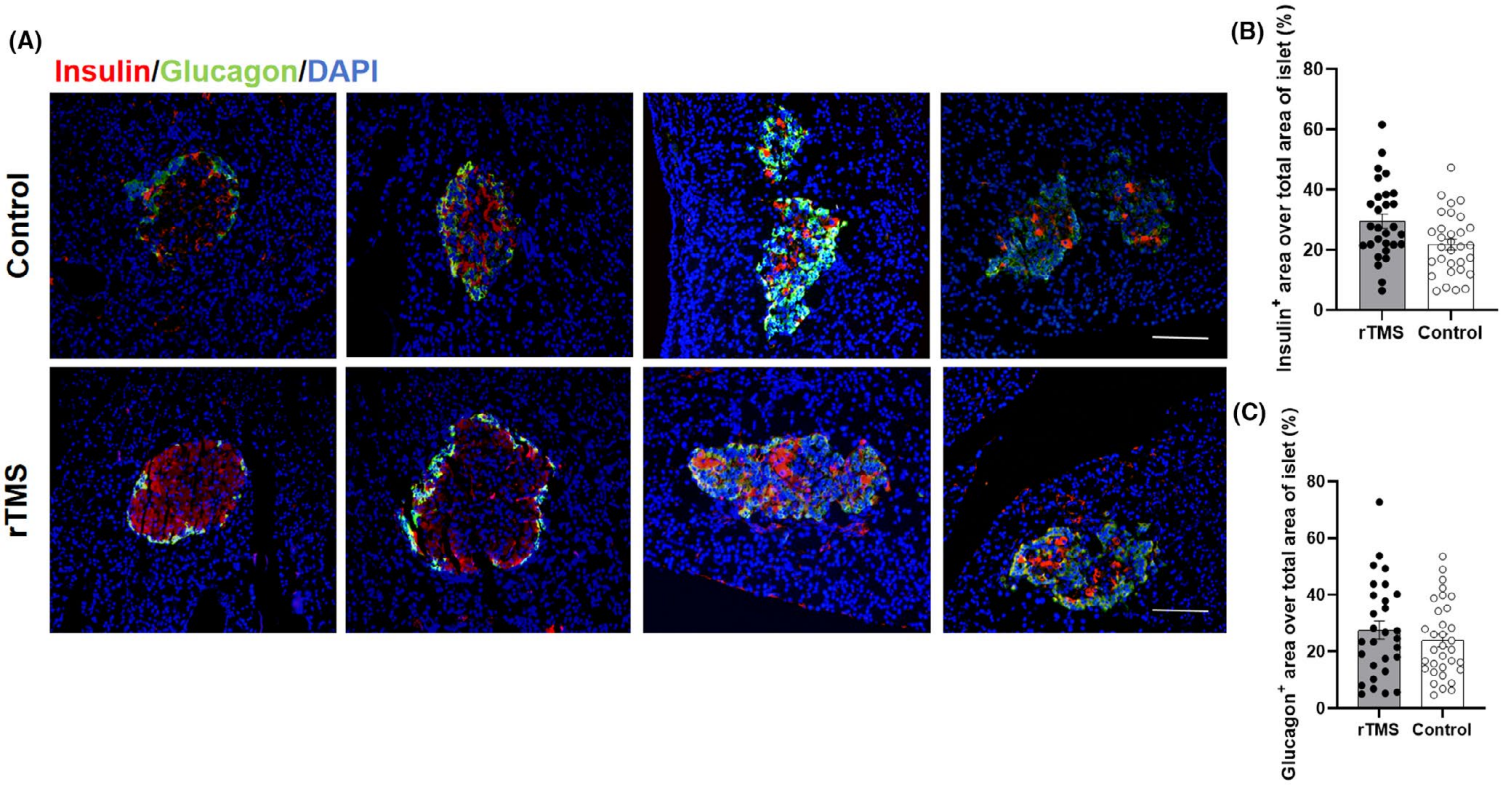
(A) Immunofluorescence staining of rat pancreas obtained from the rTMS and Control groups. Insulin β‐cells were stained red, glucagon α‐cells were stained green and the cell nuclei were stained blue by DAPI. Scale bar = 100 μm. (B) Average percentage of insulin^+^ area over total islet area. Data presented as mean ± s.e.m., *n* = 33. (C) Average percentage of glucagon^+^ area over total islet area. Data presented as mean ± s.e.m., *n* = 33.

Alterations in glucose tolerance and insulin resistance did not appear to be the result of changes in islet β‐cell mass (Figure 3). As shown by the immunofluorescence staining results of rat pancreata extracted from both experimental groups, no significant changes in insulin^+^ or glucagon^+^ fluorescence signals were seen (Figure 3B,C). Both the rTMS and Control groups had some islets that exhibited normal rodent islet morphology, with the majority of insulin‐producing β‐cells in the core (Figure 3A, shown in red, upper left two panels for Control, lower left two panels for rTMS) and glucagon‐secretion α‐cells around the edges (shown in green). A certain degree of β‐cell attrition was observed in both groups, demonstrated by diminished insulin fluorescence (Figure 3A, shown in red, upper right two panels for Control; lower right two panels for rTMS).

**FIGURE 4:**
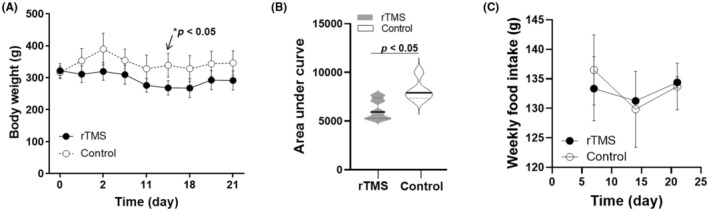
(A) Average body weight recorded for the rTMS‐treated (black solid circles) and Control (open circles) groups of T2D rats. (B) Area under the curve (AUC) representation of average body weight data of the rTMS (solid gray shaded area) and Control (gray outlined area) groups. Median values are shown as black solid lines. (C) Average weekly food intake recorded for the rTMS and Control groups. All data are presented as mean ± s.e.m., *n* = 6.

The amelioration of insulin resistance observed from the rTMS group could be due to a moderate albeit statistically significant weight reduction during the rTMS intervention period, as shown in Figure 4A,B. This is also in line with previous studies reporting weight loss in obese individuals following a 4‐week rTMS treatment.^9^ Moreover, this weight reduction appeared to be independent of food intake, as no change in weekly food intake was detected (Figure 4C). In addition, prior to rTMS treatment, the average body weight for the HFD/STZ‐induced hyperglycemic rats was significantly larger than the parallel normal‐chow‐fed rats (319.14 ± 13.33 g for the HFD/STZ‐induced hyperglycemic group and 262.67 ± 3.77 g for the normal‐chow group, *p* < 0.02), demonstrating an overweight phenotype of the HFD/STZ‐induced hyperglycemic T2D rats.

### 3.1 rTMS treatment reduced circulating lipid levels

**FIGURE 5:**
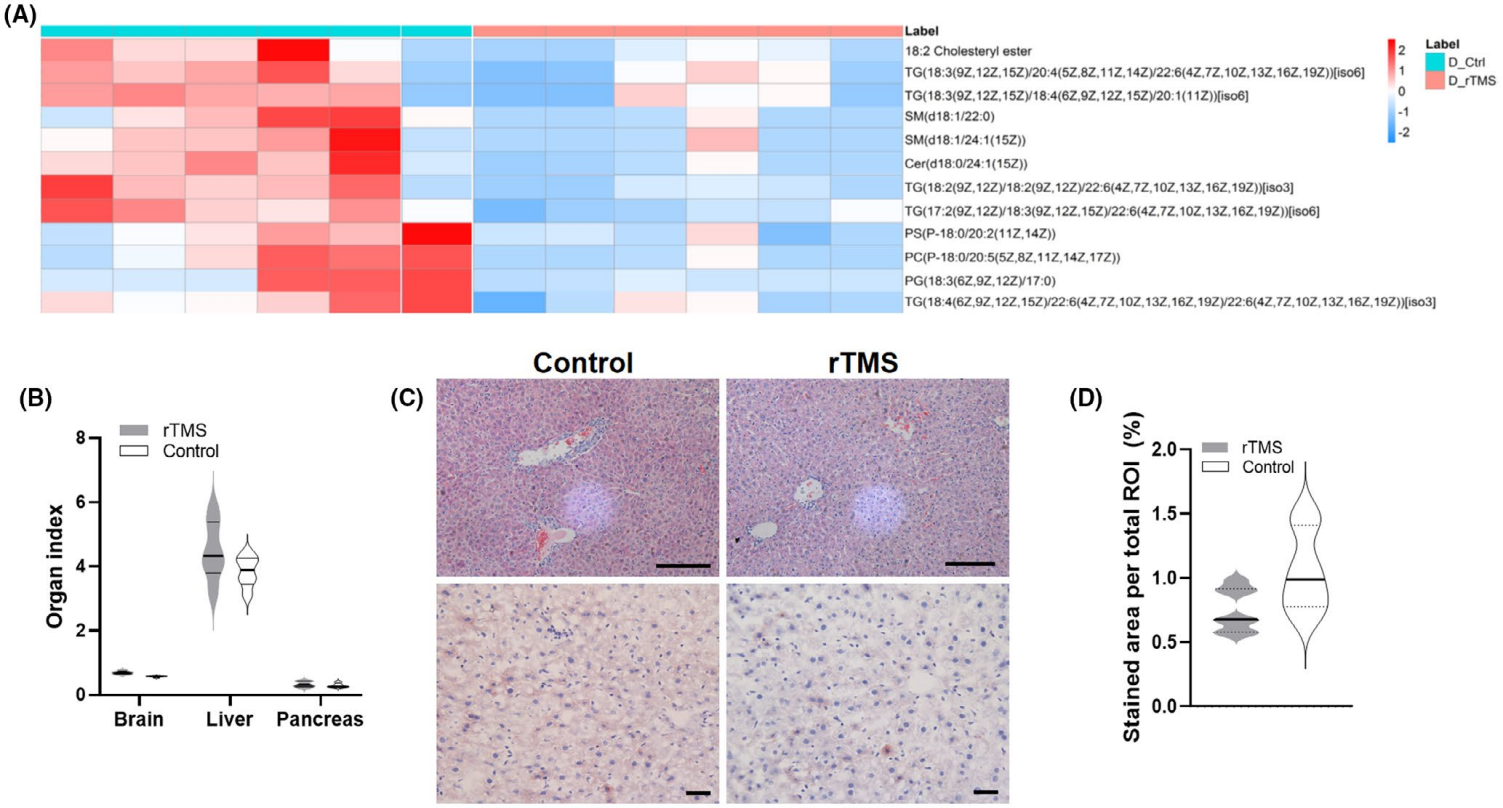
(A) Heatmap of selective lipid analogues in serum samples collected from the rTMS (orange) and Control (cyan) groups. (B) Organ index of the brain, liver and pancreas extracted from the rTMS (gray shaded area) and Control (gray outlined area) groups. Data are presented as mean ± s.e.m., *n* = 6. (C) Representative H&E (upper panels) and Oil Red O (lower panels) images of liver samples extracted from the rTMS and Control groups. Scale bar = 100 μm. (D) Average percentage of Oil Red O stained area per region of interest (ROI) of liver samples extracted from the rTMS and Control groups. Data are presented as mean ± s.e.m., *n* = 10–12.

Potential mechanisms responsible for the weight loss and improved insulin resistance were then investigated. Lipidomics was performed using serum samples collected from the rTMS‐treated and sham Control rats. As shown in Figure 5A, circulating levels of multiple lipid analogues, including the cholesteryl ester, triglyceride (TG), ceramides and selective glycerophospholipids were significantly reduced in the serum samples of the rTMS‐treated T2D rats. These results are also consistent with previous studies reporting beneficial effects of rTMS in modulating the serum lipid profile of aging humans.^15^

**FIGURE 6:**
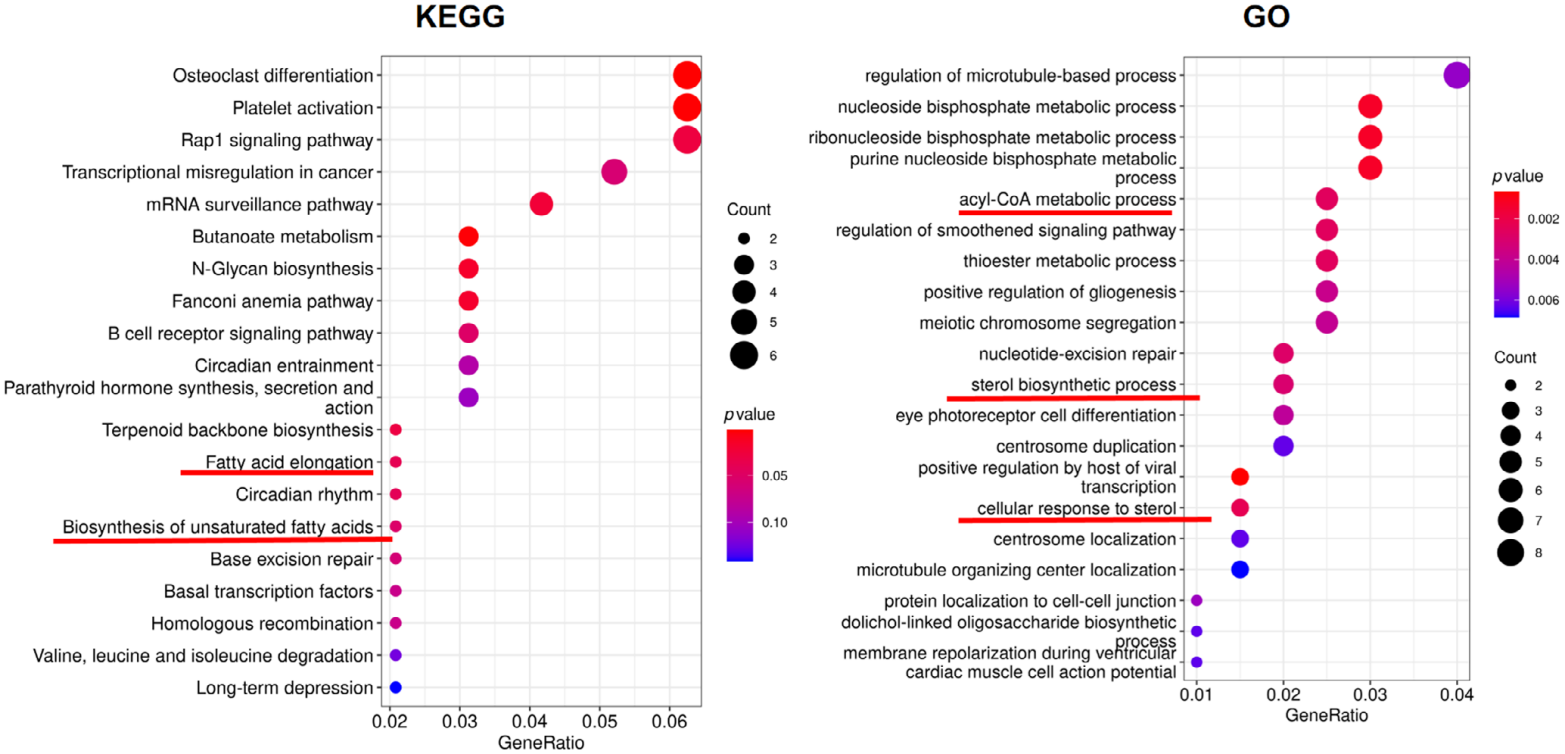
Categories of different hepatic genes using enrichment of gene ontology (GO) and KEGG pathways.

In order to examine any potential liver lipid deposition and the possible impact of rTMS in the brain and pancreas, we also measured the average weights of rat brain, liver and pancreas from both experimental groups and calculated values of organ indexes for these tissues. No significant differences were observed between the two experimental groups regarding the organ indexes of the brain, liver and pancreas (Figure 6B). In addition, no evident lipid deposition was seen in liver samples obtained from both rTMS and Control groups (Figure 5C). It was observed that although the Oil Red O staining of liver samples from the Control group appeared to be slightly darker (Figure 5C, lower panels), the overall percentages of Oil Red O staining area were not significantly different between the rTMS and Control groups (Figure 5D). The lack of hepatic morphological difference was likely due to a relatively short T2D duration. Further exploration using older rats with more advanced diabetic conditions should be considered to examine the potential impact of rTMS treatment on hepatic lipid metabolism and its underlying mechanisms.

### 3.2 rTMS affects hepatic and hypothalamic gene expression

Liver is a major metabolic organ that regulate metabolic activities, including cholesterol biosynthesis,^16^ glucose and fatty acid metabolism.^17^ It is also responsible for T2D‐related exacerbation of insulin resistance and is subject to regulation by various hormones and growth factors. In order to understand the potential mechanisms that underlie the ameliorative effects of rTMS on systemic insulin sensitivity and weight loss in T2D rats, we extracted liver and hypothalamus samples from both experimental groups at the end of the rTMS‐treated period and RNA‐seq analysis was performed.

**FIGURE 7:**
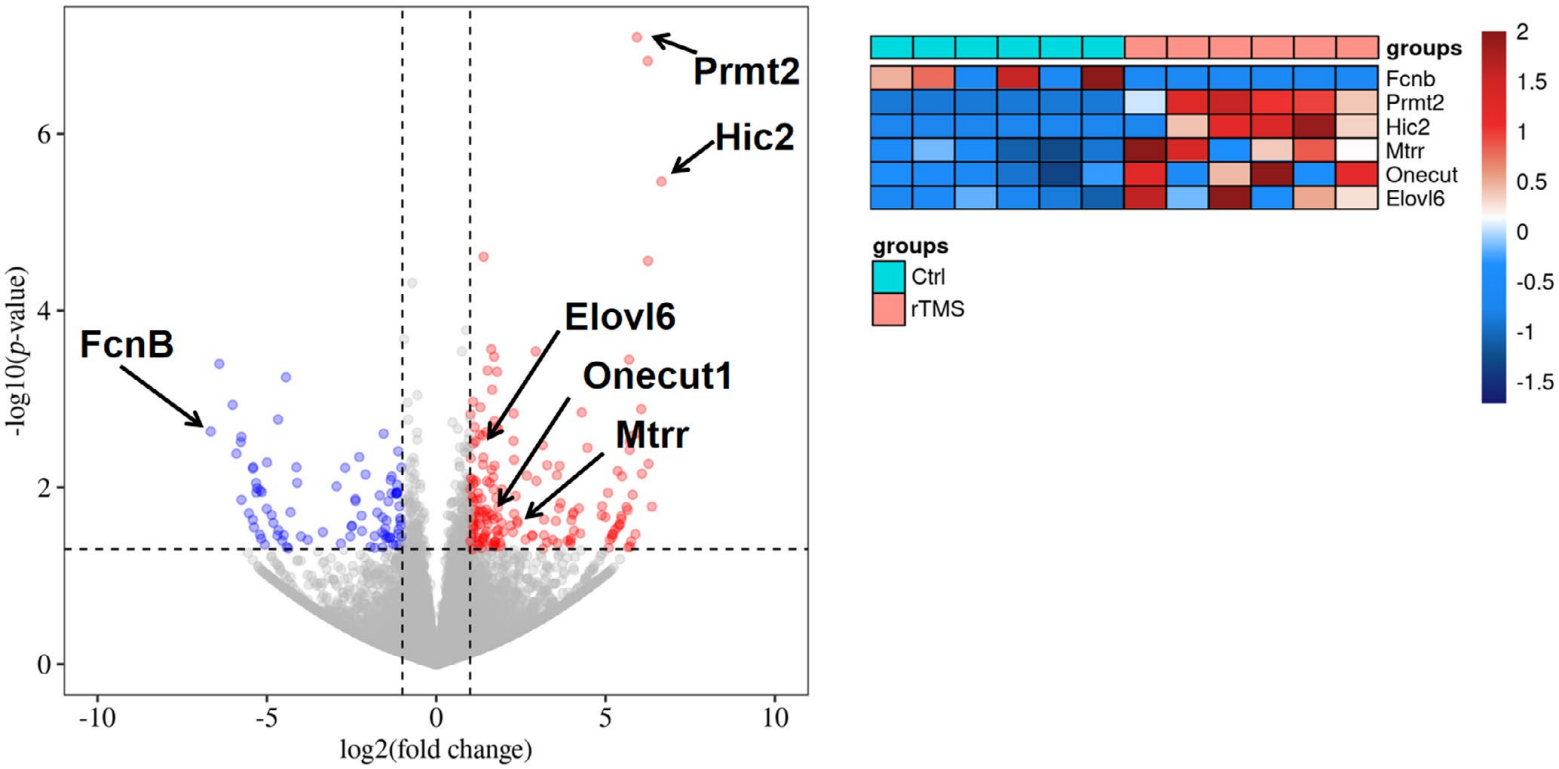
Volcano plot and heatmap of RNA‐seq analysis showing hepatic genes changed in rTMS (orange) and Control (cyan) groups. Genes of interest are highlighted, with downregulated genes shown in blue and upregulated genes in red.

As expected, results from GO and KEGG enrichment showed altered expression of hepatic genes that are involved in fatty acid metabolism as well as sterol bioactivity (Figure 7, underlined red), in line with the serum lipidomics results. Metabolism of acetyl‐CoA was also implicated. Considering the metabolic function of acetyl‐CoA both as a signaling molecule and as a regulating metabolic substrate for fatty acids and sterols,^18^ the results further suggest a regulatory potential of rTMS on hepatic fatty acid and sterol metabolism and signaling. A Volcano plot (Figure 7) specified selective hepatic genes that were significantly up‐ or downregulated following rTMS treatment. Thus, ficolin B (*fcnb*), a molecule associated with cell apoptosis and necrosis,^19^ was downregulated in the rTMS group. *Premt2*, a glucose‐sensitive factor that is involved in the regulation of cholesterol efflux, was upregulated in the rTMS‐treated rats. It has also been shown to be associated with atherosclerosis in diabetic individuals.^20^ Another vascular‐related multi‐faceted transcription factor, *Hic2*, was also found to be upregulated following rTMS. Indeed, *Hic2* has been reported to participate in multiple biological processes including, among others, energy metabolism and metabolic diseases, suggesting a diverse roles of rTMS.^21^, ^22^ In addition, *mtrr*, also found to be upregulated in the rTMS group, has been implicated in hepatic fatty acid metabolism and energy storage,^23^ supporting the results obtained from serum lipidomics.

**FIGURE 8:**
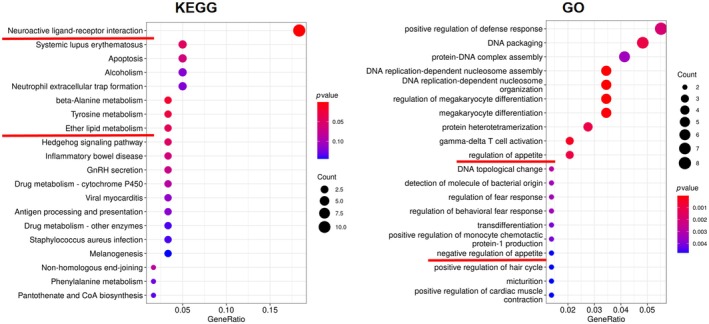
Categories of different hypothalamic genes using enrichment of gene ontology (GO) and KEGG pathways.

Diabetes‐associated gene expression was also observed, for example, the upregulation of hepatic expression of the One Cut Homeobox 1 (*onecut1*; Figure 8). As reported recently, the homo‐ and heterozygous *onecut1* mutation alone could result in endocrine dysfunction and multi‐organ metabolic imbalance, causing onset of multiple types of diabetes.^24^ In addition, expression of *elovl6*, a potential regulator of obesity, insulin sensitivity and hepatosteatosis, was shown to be increased in the rTMS‐treated rat liver. Since knockout *of elovl6* resulted in an obese phenotype and exacerbation of hepatic steatosis, our results once again support the positive metabolic regulatory effects of rTMS in T2D.^25^

**FIGURE 9:**
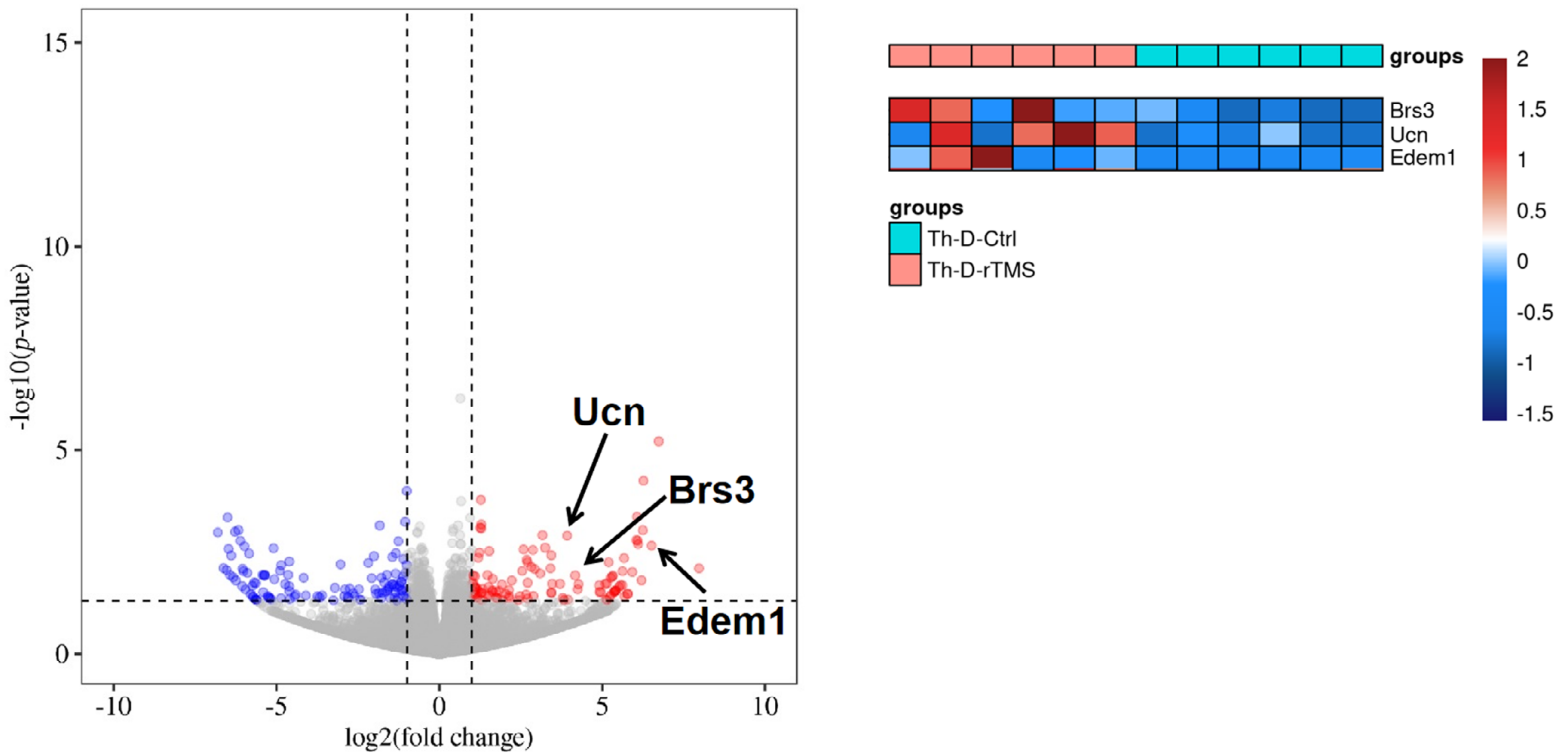
Volcano plot and heatmap of RNA‐seq analysis showing hypothalamic genes changed in rTMS (orange) and Control (cyan) groups. Genes of interest are highlighted, with downregulated genes are shown in blue and upregulated genes in red.

RNA‐seq analysis was also performed using hypothalami obtained from both experimental groups (Figures 8 and 9). Similar to previously reported human studies,^9^, ^10^ rTMS elicited expression of genes that are involved in appetite regulation, such as *ucn*, which is associated with decreased food intake following administration.^26^ Regulatory genes implicated in neuroactivity were also involved, which is expected since rTMS is known to affect cerebral neural activities.^27^ Moreover, a changed expression pattern of genes that are associated with ether lipid metabolism was also observed (Figure 8). Considering the involvement of ether lipids in metabolic diseases including obesity, nonalcoholic steatohepatitis and nonalcoholic fatty liver disease,^28^ rTMS‐elicited central regulation of ether lipid metabolism could potentially lead to peripheral fatty acid biosynthesis and signaling, as already implicated by data from the serum lipidomics (Figure 5) and RNA‐seq results (Figures 6 and 7).

Specifically, the bombesin‐like receptor 3 (*brs3*), an orphan receptor gene, was upregulated in the rTMS group. Lack of function of Brs3 has been reported to lead to a decrease in metabolic rate and body temperature.^29^, ^30^, ^31^ Moreover, expression of *edem*, a cell mediator that is involved in insulin signaling and maintenance of metabolic homeostasis,^32^ was also elevated, adding to the evidence in support of a potential role of rTMS on energy metabolism.

## 4 DISCUSSION

Insulin resistance is often considered an important pathological manifestation of T2D. Physiologically, the systemic metabolism and overall daily functioning are dynamically maintained by sensitivity to insulin in various tissue types including the skeletal muscles, liver, adipose tissue, and the central nervous system. Insulin resistance may be the cause or the result of glucose intolerance in the pre‐diabetic or diabetic state, and has been a pharmacological target for glucose management. Current anti‐diabetics such as metformin and thiazolidinediones all exert their physiological glucose lowering benefit by improving insulin sensitivity.^33^, ^34^

The central nervous system is also a major glucose‐consuming organ that is responsive to insulin action. On the other hand, the role of the central nervous system in systemic metabolic regulation has also been demonstrated. Indeed, the metabolic action of insulin as well as body weight have been shown to be mediated by insulin receptors expressed in the hypothalamus.^35^ Research has also shown that enhancing insulin action within these brain regions can regulate peripheral metabolism, while inhibition of insulin receptors in specific regions of the hypothalamus led to impaired insulin secretion and glucose intolerance.^11^

**FIGURE 10:**
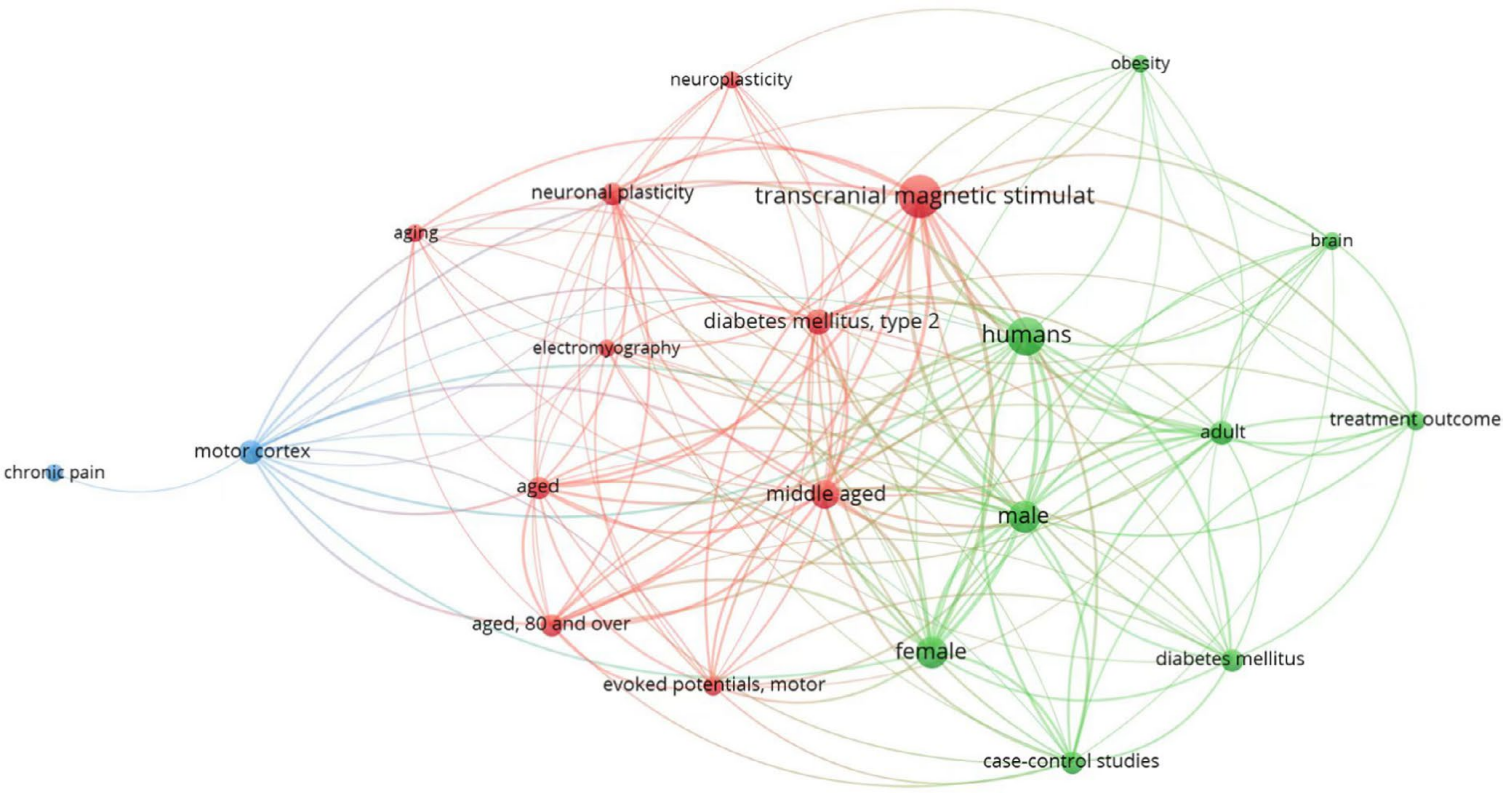
Pubmed reference analysis showing associations among research areas using the keywords transcranial magnetic stimulation and diabetes. Articles published in the last 20 years (ranging from 2003.11 to 2023.11) were collectively searched and analyzed.

Given that the keyword “rTMS” is strongly associated with obesity, diabetes, T2D and aging in a Pubmed literature search (Figure 10), the present study aimed to investigate the potential effects of manipulation of the central nervous system via high‐frequency rTMS on systemic metabolism in a diseased condition using HFD/STZ‐induced T2D rats. We observed that following a 10‐day treatment with high‐frequency rTMS, parameters including average body weight, fasting plasma glucose, glucose tolerance, and insulin sensitivity all showed significant improvement in T2D rats. Following a 21‐day period of rTMS administration, weight loss and amelioration of insulin resistance were maintained, accompanied by decreased circulating levels of the cholesteryl ester, TG and ceramides. Further RNA‐seq analyses revealed upregulation of genes that are related to diabetes, obesity, fatty acid synthesis and appetite in the liver as well as in the hypothalamus, suggesting a multi‐faceted signaling mechanism that links the central‐peripheral axis of the rTMS, which requires future investigation.

In addition, our present data are also in line with previous studies reporting the positive impact of rTMS on metabolic parameters.^33^, ^34^ Thus, it has been shown that repetitive high‐frequency rTMS exerted positive effects on local glucose utilization in the cerebral regions of rats,^36^ healthy individuals,^37^ and in individuals with stroke or depression,^36^, ^37^, ^38^, ^39^ all of which are consistent with our results observing increased expression of hypothalamic genes that are involved in neural activity (Figure 7). More relevantly, studies using low‐voltage transcranial direct current stimulation (tDCS) observed improved glucose tolerance of healthy male individuals,^40^, ^41^ although the effect of tDCS on individuals with pathological conditions are unknown. In the present study, high‐frequency rTMS in intermittent theta cluster mode (iTBS) was selected. This is because the rhythm of pulse transmission determines the effectiveness of rTMS,^42^ and more importantly, compared with just rTMS, the iTBS mimics the high‐frequency pulse sequence of neuronal discharge, and is comparatively safer and more effective.^43^, ^44^ And indeed, using T2D rats, we show here that iTBS rTMS stimulation elicited improvements in systemic insulin sensitivity accompanied by weight loss, suggesting its potential as interventional therapeutic approach for T2D.

Previous studies have shown that maintaining physiological redox homeostasis could be a possible mechanism via which electromagnetic stimulation regulates plasma glucose levels.^45^ Indeed, it has long been known that reactive oxygen species (ROS) play an important role in systemic as well as cellular metabolism.^46^, ^47^ Excessive levels of ROS often lead to oxidative stress, which negatively affects the insulin signaling pathways.^48^ To provide further insight, a recent study combining static magnetic and electric fields (vertical electrostatic field, 7 kV/m; horizontal static magnetic field, 3 mT; sBE), using T2D mice, demonstrated that sBE improved insulin sensitivity and plasma glucose levels by reducing oxidative stress.^49^ In addition, another study involving application of a static magnetic field (sMF) to HFD/STZ‐induced T2D mice showed that vertical downward sMF with an intensity of 100 mT could effectively ameliorate hyperglycemia, while also reducing weight gain, tissue damage and liver steosis, supporting the evidence that electromagnetic fields can regulate metabolic parameters by restoring systemic redox balance.^50^ These results, together with the results presented in the current study, implicate the potential mechanisms for the effects of high‐frequency rTMS on insulin sensitivity and weight reduction in T2D rats, but further investigation of the mechanisms is still needed.

## 5 CONCLUSION

We report here evidence of iTBS rTMS stimulation as a potential interventional therapeutic option for T2D. Significant improvements in insulin sensitivity accompanied by weight loss were observed in T2D rats that received rTMS treatment compared to controls. The beneficial effects could possibly be the result of a central‐to‐peripheral regulatory action of rTMS on hepatic lipid metabolism, which is worth further exploration.

## AUTHOR CONTRIBUTIONS

**Xuanjin Chen:** Data curation; formal analysis; investigation; methodology. **Ruru Wang:** Data curation; investigation; methodology. **Xin Wang:** Conceptualization; investigation; methodology; supervision. **Ming Liu:** Resources; software; validation. **Zhipeng Liu:** Conceptualization; resources; supervision. **Tao Yin:** Conceptualization; resources; supervision. **Chen Li:** Conceptualization; funding acquisition; resources; supervision; writing – original draft.

## FUNDING INFORMATION

We are grateful for the generous financial support of the National Key Research and Development Programme of China (No. 2020YFA0803701), the CAMS Innovation Fund for Medical Sciences (2021‐12M‐1‐052) and the National Natural Science Foundation of China (no. 81973699, no. 82274361, and no. 52107241).

## CONFLICT OF INTEREST STATEMENT

The authors declare no conflicts of interest.

## ETHICS STATEMENT

The protocols involved in the animal experiments performed in this study were designed and approved by the Animal Ethics and Welfare Committee of the Chinese Academy of Medical Sciences (Approval No. IRM‐DWLL‐2020001) and performed in strict accordance with the Guidelines for Use and Care of Laboratory Animals of the Chinese Academy of Medical Sciences.
